# Theoretical and Experimental Studies of Permeate Fluxes in Double-Flow Direct-Contact Membrane Distillation (DCMD) Modules with Internal Recycle

**DOI:** 10.3390/membranes16010037

**Published:** 2026-01-06

**Authors:** Chii-Dong Ho, Ching-Yu Li, Thiam Leng Chew, Yi-Ting Lin

**Affiliations:** 1 Department of Chemical and Materials Engineering, Tamkang University, Tamsui, New Taipei 251301, Taiwan; 2 Department of Chemical Engineering, Faculty of Engineering, Universiti Teknologi PETRONAS, Seri Iskandar 32610, Perak, Malaysia; thiamleng.chew@utp.edu.my; 3 CO2 Research Center (CO2RES), Institute of Contaminant Management, Universiti Teknologi PETRONAS, Seri Iskandar 32610, Perak, Malaysia

**Keywords:** permeate flux, seawater desalination, flat-plate DCMD module, double-flow operation, internal recycle

## Abstract

A new DCMD module design that introduces an insulation barrier of negligible thickness to divide the open duct of the hot-saline feed into two subchannels for dual-flow operation was investigated. This configuration enables one subchannel to operate in a cocurrent-flow mode and the other in a countercurrent-flow recycling mode, thereby significantly enhancing the permeate flux. Theoretical and experimental investigations were conducted to develop modeling equations capable of predicting the permeate flux in DCMD modules. These studies demonstrated the technical feasibility of minimizing temperature polarization effects while improving flow characteristics to boost permeate flux. Results indicated that increasing both convective heat-transfer coefficients and residence time generally improved device performance. The dual-flow operation increased fluid velocity and extended residence time, leading to reduced heat-transfer resistance and enhanced heat-transfer efficiency. Theoretical predictions and experimental results consistently showed that the absorption flux improved by up to 40.77% under the double-flow operation with internal recycling configuration compared to a single-pass device of identical dimensions. The effects of inserting the insulation barrier on permeate flux enhancement, power consumption, and overall economic feasibility were also discussed.

## 1. Introduction

The membrane distillation (MD) process is a promising option for applications such as desalination, solution concentration, and wastewater treatment [1]. The direct-contact membrane distillation (DCMD) modules were operated at moderate temperatures ranging from 45 °C to 60 °C using a hot-seawater feed stream and a constant cold inlet stream. This setup allowed water vapor to pass through the membrane while retaining salts and impurities. Owing to its simplicity and comparatively low energy requirements [2], MD can be economically feasible. The technology also addresses several shortcomings of conventional packed-column absorption processes—such as entrainment, flooding, channeling, and foaming [3]. In direct-contact membrane distillation (DCMD), vapor–liquid equilibrium is established at the hot feed–membrane interface where volatile species vaporize and are driven across a hydrophobic microporous membrane by a vapor-pressure gradient [4] and then condense on the cold side to produce high-purity water [5]. The temperature gradient established during the DCMD operation caused interactions between the membrane surface and bulk fluid temperatures, leading to a temperature difference that induced significant thermal energy loss due to temperature polarization effects. An assessment of the membrane effectiveness was conducted to determine the permeate flux improvement that balanced a desirable increase in heat transfer coefficient and undesirable energy consumption. The evaluation concluded that the temperature effect had been identified as a significant limit for the MD flux performance, which is the major factor governing permeate-flux performance [6], particularly at the industrial scale [7]. Detailed modeling studies are required to help identify and simulate the magnitude of these effects on the MD system. Overall, the implementation of a double-flow channel exhibits significant potential for substantially augmenting permeate flux in the DCMD module due to mitigation of the thermal boundary layer, with a smaller thermal resistance and temperature polarization effect as well. It is possible to reduce the temperature polarization effect as well as to increase the rate of mass transfer by improving the flow channels [8] in DCMD modules or other commercial-scale pilots and applications, such as vacuum membrane distillation modules [9] and reverse osmosis [10].

Recent research on DCMD has heavily focused on heat-transfer enhancement and DCMD module design to combat the primary limitation of temperature polarization and boost energy efficiency. The integration of UV-assisted grafting for membrane surface modification has also been explored as a method to improve performance parameters [11]. Furthermore, advancements have been made with innovative membrane materials and configurations, such as Janus membranes and membranes modified with photothermal materials [12], which enable localized “self-heating” near the membrane surface to harness localized heating effects, substantially mitigating temperature polarization and leading to marked increases in both flux and thermal efficiency. This concerted effort on novel module and material development highlights the drive to push DCMD towards higher productivity and thermal performance. A major research thrust is the development of innovative DCMD module designs and membrane materials to enhance heat transfer and maximize permeate flux. The core challenge in DCMD remains the large temperature gradient across the boundary layer, suffering from temperature polarization near the membrane surface, which reduces the effective temperature driving force and thus diminishes permeate flux [13]. To suppress the boundary-layer thickness formed by polarization between the bulk stream and the membrane surface, numerous studies have investigated innovative module designs, particularly by incorporating turbulence promoters and spacers into the flow channels, such as roughened surfaces [14] and carbon-fiber spacer channels [15]. For instance, the use of turbulence promoters with varying hydraulic diameters or geometrically shaped promoters has been shown to disrupt the thermal boundary layer, increase the convective heat-transfer coefficient, and significantly enhance permeate flux [16,17]. Increasing turbulence intensity to mitigate temperature polarization has been shown to enhance the mass-transfer coefficient [18]; nevertheless, the accompanying pressure drop in the feed channel must also be evaluated for economic feasibility.

A key focus for improving energy efficiency and achieving high water recovery is the use of recycle-enhanced DCMD modules and system-level optimization strategies that leverage heat recovery. The concept of operating in continuous recirculation mode is frequently analyzed. This process reveals that an inherent trade-off exists between these two performance indicators, the cold flow rate and module dimensions—which are highly sensitive and configuration-dependent [19]. A promising optimization strategy that allows DCMD to effectively recover heat from brine achieves high water recovery and reduces the overall energy footprint of the combined process [20]. The evolution of DCMD technology is underpinned by sophisticated modeling frameworks and optimization strategies crucial for industrial scalability. Traditional mechanistic models, which couple heat and mass transfer, are being supplemented and even outperformed by machine-learning approaches in predicting permeate flux by considering complex input parameters, offering a powerful tool for robust performance prediction [21,22].

In terms of system-level optimization, the focus is on maximizing water productivity while minimizing thermal energy demand. The strategy of implementing recycle-enhanced DCMD modules is repeatedly evaluated. While brine recycling is essential for achieving high water recovery and utilizing waste heat, research cautions that it must be carefully controlled, as increased thermal energy input is required to heat the larger recycle streams [23]. A strategy for designing and operating heat- and mass-transfer equipment with external or internal reflux can effectively increase fluid velocity and enhance heat-transfer coefficients, thereby improving device performance [24,25]. Heat-transfer problems in closed conduits with end-recycle have also been investigated [26]. Though the mechanistic analysis of mass transfer in the present study could be mostly analogized from that of heat exchangers in our previous works [27,28], the manners of transport across the hydrophobic membrane are somewhat different. Performance improvement through double-flow operation was found to increase with both the volumetric flow rate and inlet saline temperature. Compared with single-pass flow channels commonly used in separation modules and reactors—such as loop reactors [29], air-lift reactors [30], and draft-tube bubble columns [31]—this approach can significantly improve heat-transfer efficiency. This paper presents theoretical predictions and experimental results for permeate flux in parallel-plate MD modules equipped with double-flow operation. Double-flow modules were tested over various volumetric flow rates and inlet saline temperatures to assess how increased convective heat-transfer in the saline feed stream affects performance, with the aim of identifying operating conditions that achieve gains with relatively modest power-consumption increments. Prior studies have reported that increasing convective heat-transfer coefficients via double-flow configurations can improve heat-transfer rates [25] while also imparting additional pressure drop in the feed channel [32]. Nevertheless, in some heat-exchanger applications, the advantages of double-flow operation may be offset by these drawbacks.

The purpose of this work is to design a new module by inserting an impermeable sheet to divide an open duct into two channels and to investigate the permeate flux improvement in double-flow DCMD modules with internal recycle. The present implementation of double-flow schemes elucidates their hydrodynamic influence on heat-transfer mechanisms, demonstrates technical feasibility, and significantly enhances permeate flux. The objective of this work is to investigate how internal recycle affects permeate-flux performance in a double-flow DCMD module in which the hot-saline feed is divided into two subchannels: one operating cocurrently and the other serving as a countercurrent internal-reflux subchannel. Applications of such devices with internal recycle have led to improved performance in membrane distillation modules. An exact solution to the problem on double-flow DCMD modules with internal refluxes is demonstrated. Therefore, the same method can be applied easily to solve other multi-stream problems in heat- and mass-transfer devices. It is believed that the availability of such a simplifying mathematical formulation for double-flow flat-plate membrane modules as developed herein adds value for analysis and design, and the same procedure can be adapted to other membrane separation processes. A strong agreement between theoretical predictions and experimental observations confirmed the reliability of the proposed design.

## 2. Membrane Distillation Apparatus and Materials

**Figure 1:**
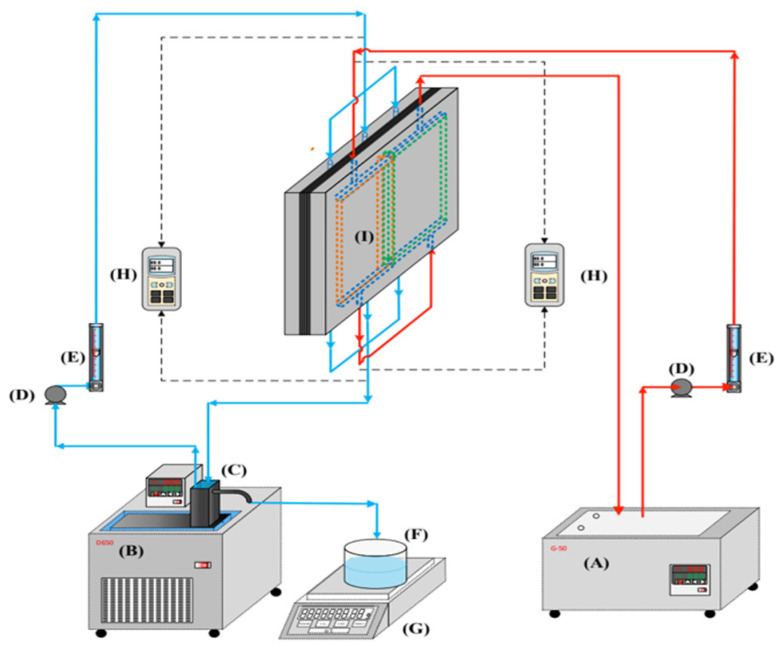
Scheme of a setup used in experiments of double-flow flat-plate DCMD modules (A—hot-fluid thermostatic tank; B—cold-fluid thermostatic tank; C—overflow barrel; D—pump; E—flow meter; F—beaker; G—electronic balance; H—temperature indicator; I—the DCMD module). Red arrow line of hot saline feed stream and blue arrow line of cold feed stream.

**Figure 2:**
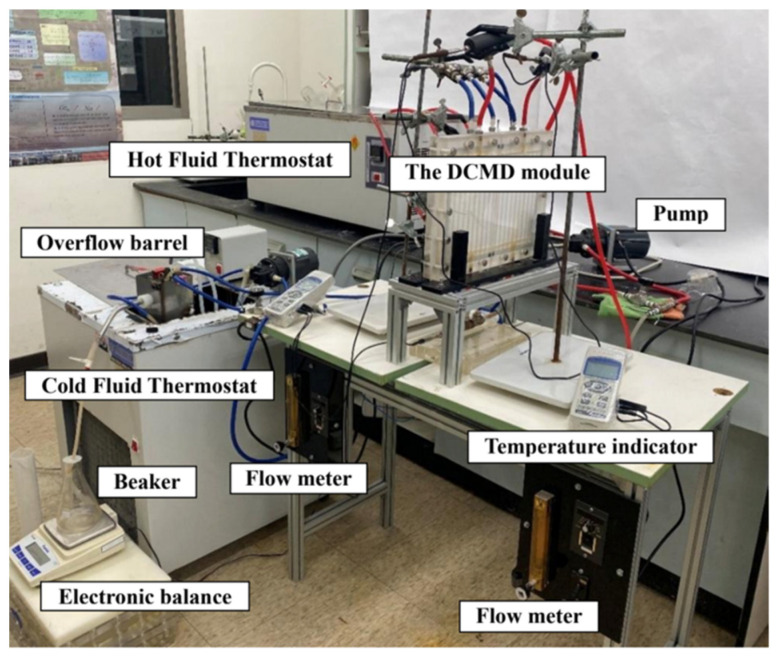
A photo of the experimental apparatus of the double-flow DCMD system.

The schematic configuration of an acrylic parallel-plate channel—length 21 cm, width 29 cm, and 2 mm channel height for each cold and hot stream—is shown in Figure 1, and a photo of the operating experimental apparatus is provided in Figure 2, where the acrylic plates are stacked as the outer walls of the parallel-plate channel.

**Figure 3:**
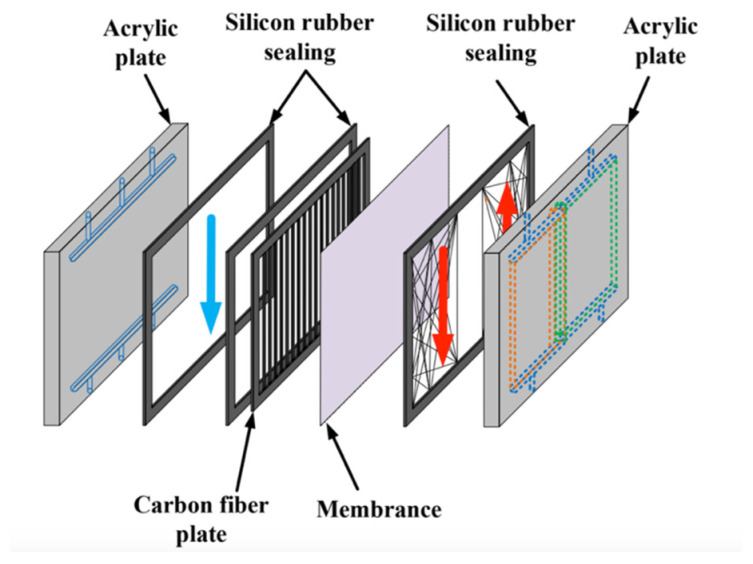
Components of the double-flow flat-plate DCMD module. Red arrow line of the hot saline feed stream and blue arrow line of the cold feed stream.

The hydrophobic membrane surfaces were supported by winding a 0.1 mm nylon fiber on the hot-saline side and inserting cross carbon-fiber spacers on the cold side, as shown in Figure 3, to prevent membrane bending and wrinkling. The spacer structure is also displayed in Figure 3. A silicone rubber gasket with a thickness of 2 mm was bonded to the acrylic plate to define the spacer channel and prevent leakage. The commercial membrane used was polytetrafluoroethylene (PTFE), supported by a polypropylene (PP) net (J020A330R, Toyo Roshi Kaisha, Ltd., Tokyo, Japan). Its principal characteristics, as specified by the manufacturer, are nominal pore size 0.2 µm, porosity 0.72, and total thickness 130 µm (PTFE 98 µm and PP 32 µm). The permeate collected as distillate was weighed using an electronic balance (XS 4250C, Precisa Gravimetrics AG, Dietikon, Switzerland). To ensure stable thermal conditions during the process, two thermostats (G-50, 60 L, 3500 W; DENG YNG, Kaohsiung, Taiwan) were utilized to supply heat, while conventional pumps (51K40RA-A, ASTK, New Taipei, Taiwan) circulated the fluid to maintain steady inlet temperatures. The system’s thermal performance was monitored using thermometer probes (TM-946, Lutron, New Taipei, Taiwan) positioned at the inlet and outlet of the flat-plate membrane modules. Experimental runs were conducted by controlling various flow rates of artificial saline water (3.5 wt% NaCl), specifically adjusted to 6.67 × 10^−6^, 8.33 × 10^−6^, 11.7 × 10^−6^, and 15.0 × 10^−6^ m^3^ s^−1^ (say 0.3, 0.5, 0.7, 0.9 L min^−1^), respectively, at inlet hot-side temperatures of 45, 50, 55, and 60 °C, with the cold stream maintained at 25 °C. Precise regulation of the saline-feed volumetric flow rates was achieved through a combination of a controller (US-2000-40W, ASTK, New Taipei, Taiwan) and dedicated flow meters (FE-091312-D for the hot stream and FN-0423112-F for the cold stream; Fong-Jei, Hsinchu, Taiwan).

**Figure 4:**
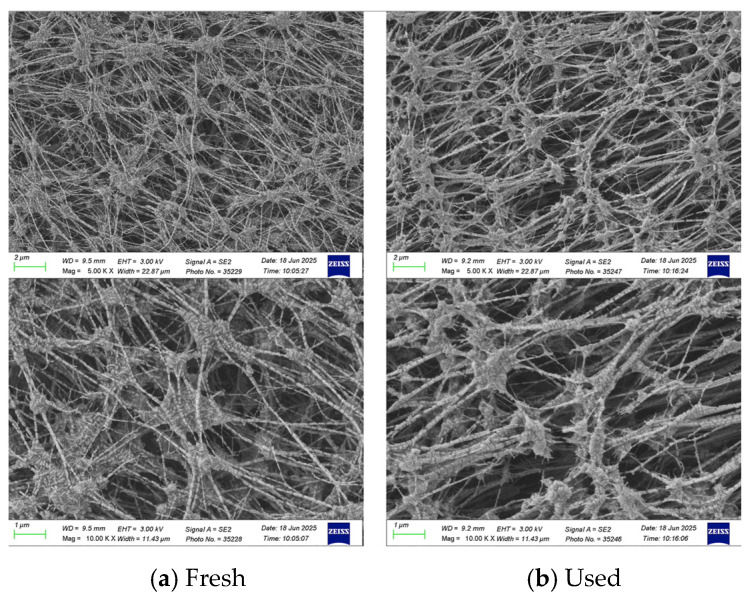
SEM images of the PTFE/PP membrane for fresh and used membranes of experimental runs.

**Figure 5:**
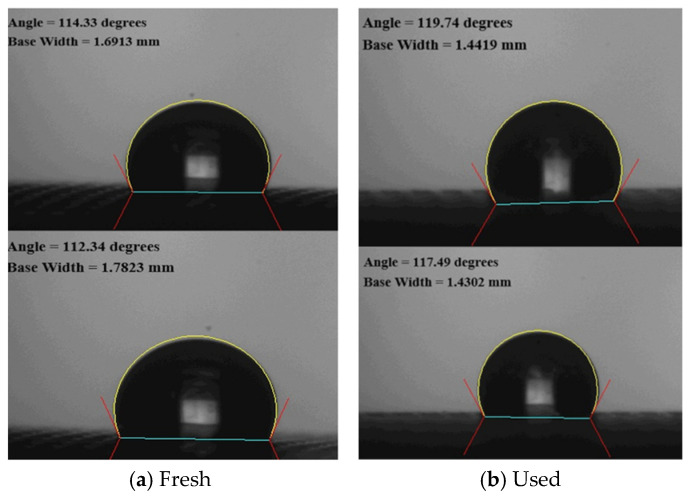
Sessile-drop contact angles of PTFE/PP membranes.

The morphology and water contact angles of the PTFE/PP composite membranes were characterized before and after experimental runs. Figure 4 illustrates the morphology of both fresh and used membranes, obtained using Scanning Electron Microscopy (SEM, Zeiss Sigma 300, Jena, Germany). To confirm surface hydrophobicity, water contact angles were measured using a First Ten Angstrom FTA-125 (Portsmouth, RI, USA). As shown in Figure 5, the contact angles ranged from 112 to 119° (116 ± 3.0°), confirming the hydrophobic nature of the membrane.

## 3. Mathematical Formulations

### 3.1. Heat and Mass Transfers

The modeling framework characterized the temperature gradient between the hot-saline and cold streams by analyzing heat-transfer resistances rooted in enthalpy-flow conservation and vapor diffusion within the module, alongside the corresponding mass-transfer behavior. To predict the permeate flux [33], the model employed the product of the transmembrane vapor-pressure difference (Δ*P*) and the membrane permeation coefficient ($c_{m}$), utilizing an expression specifically designed for microporous hydrophobic membranes [34].

(1) $$\begin{array}{c} N^{\prime \prime }=c_{m}\Delta P=c_{m}\left[P_{1}^{sat}(T_{1})-P_{2}^{sat}(T_{2})\right]\\ \;\;{=c}_{m}{\left.\frac{dP}{dT}\right|}_{T_{m}}\left(T_{1}-T_{2}\right)=c_{m}\frac{P_{m}\lambda M_{w}}{RT_{m}^{2}}\left(T_{1}-T_{2}\right) \end{array}$$

and

(2) $$c_{m}={\left(\frac{1}{c_{K}}+\frac{1}{c_{M}}\right)}^{-1}={\left\{{\left[\frac{2\sqrt{8\pi }}{3}\frac{\varepsilon \;r}{\tau \delta_{m}}{\left(\frac{M_{w}}{RT_{m}}\right)}^{1/2}\right]}^{-1}+{\left[{\left|Y_{m}\right|}_{ln}\frac{D_{m}\varepsilon }{\delta_{m}\tau }\frac{M_{w}}{RT_{m}}\right]}^{-1}\right\}}^{-1}$$

in which

(3) $${\left|Y_{m}\right|}_{ln}=\frac{\left(P_{T}-P_{2}\right)-\left(P_{T}-P_{1}\right)}{P_{T}ln\left[\frac{\left(\left(P_{T}-P_{2}\right)\right)}{\left(\left(P_{T}-P_{1}\right)\right)}\right]},\;\tau =\frac{1}{\varepsilon },\;D_{m}=\frac{1\times 10^{-7}T_{m}^{1.75}}{\frac{P_{T}}{101,325}M_{w-air}^{0.5}{\left(\sigma_{w}^{\frac{1}{3}}+\sigma_{air}^{\frac{1}{3}}\right)}^{2}},M_{w-air}={\left[\left(\frac{1}{M_{w}}\right)+\left(\frac{1}{M_{air}}\right)\right]}^{-1}$$

**Figure 6:**
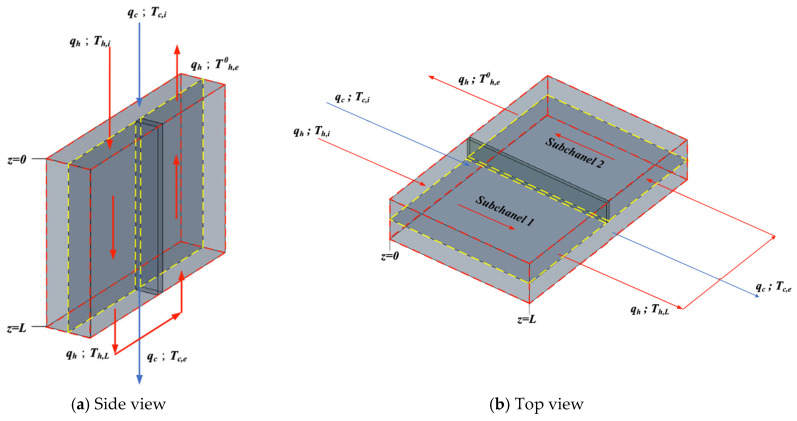
Flat-plate membrane distillation module with internal recycle.

Water vapor diffuses exclusively through the porous hydrophobic membrane in the membrane distillation process, which the bulk temperatures of the feed streams denoted as $T_{h,1}$ and $T_{h,2}$ (both subchannels of the hot-saline feed stream) and $T_{c}$ (the cold feed stream), and the corresponding membrane surface temperature as $T_{hm}$. Regarding Figure 6, one obtains

(4a) $$q_{h,1}^{\prime \prime }=\frac{1}{2}h_{h,1}\left(T_{h,1}-T_{hm}\right),\;\text{left-hand-side}\;\mathrm{subchannel}$$

(4b) $$q_{h,2}^{\prime \prime }=\frac{1}{2}h_{h,2}\left(T_{h,2}-T_{hm}\right),\;\text{right-hand-side}\;\mathrm{subchannel}$$

(5) $$q_{m}^{\prime \prime }=\left[N^{\prime \prime }\lambda +\frac{k_{m}}{\delta_{m}}\;(T_{hm}-T_{cm})\right]=H_{m}\left(T_{hm}-T_{cm}\right),\;\mathrm{membrane}\;\mathrm{region}$$

(6) $$q_{c}^{\prime \prime }=h_{c}(T_{cm}-T_{c}),\;\mathrm{cold}\mathrm{feed}\mathrm{stream}$$

where N″λ is categorized as the latent heat of vaporization. 

A fundamental requirement of this model is the energy balance across the system, where heat transfer across the hot stream, membrane, and cold stream must equalize according to Equations (4a)–(6) ($q_{h,1}^{\prime \prime }+q_{h,2}^{\prime \prime }=q_{m}^{\prime \prime }$ and $q_{m}^{\prime \prime }=q_{c}^{\prime \prime }$).

(7) $$\frac{1}{2}h_{h,1}\left(T_{h,1}-T_{hm}\;\right)+\frac{1}{2}h_{h,2}\left(T_{h,2}-T_{hm}\;\right)=H_{m}\left(T_{hm}-T_{cm}\right)=h_{c}(T_{cm}-T_{c}\;)$$

Solving Equation (7) with respect to $T_{h,1}$, $T_{h,2}$ and $T_{c}$, one obtains

(8) $$T_{h,1}=T_{hm}+\frac{H_{m}}{h_{h,1}}(T_{hm}-T_{cm})$$

(9) $$T_{h,2}=T_{hm}+\frac{H_{m}}{h_{h,2}}(T_{hm}-T_{cm})$$

(10) $$T_{c}=T_{cm}-\frac{H_{m}}{h_{c}}(T_{hm}-T_{cm})$$

Additionally, the temperature polarization effect [33] on the device performance of the DCMD module, which defines the thermal boundary-layer resistance capable of limiting flux, was quantified using temperature polarization coefficients ($\gamma_{m,1}\;and\;\gamma_{m,2}$).

(11) $$\gamma_{m,1}=\frac{\left(T_{hm}-T_{cm}\right)}{\left(T_{h,1}-T_{c}\right)}=\frac{h_{h,1}h_{c}}{h_{h,1}h_{c}+h_{h,1}H_{m}+h_{c}H_{m}}$$

(12) $$\gamma_{m,2}=\frac{\left(T_{hm}-T_{cm}\right)}{\left(T_{h,2}-T_{c}\right)}=\frac{h_{h,2}h_{c}}{h_{h,2}h_{c}+h_{h,2}H_{m}+h_{c}H_{m}}$$

### 3.2. Theory and Analysis

Figure 6 shows the heat exchanger operated first in cocurrent flow and then followed by a countercurrent-flow recycle. An insulated plate of negligible thickness is placed normal to the hot-saline feed stream along the channel centerline to divide it into two subchannels of equal width, w/2, and a pump is installed to enable internal recycle. Thus, in the hot-saline feed stream, the inlet fluid—with time rate of heat capacity $m_{h}\;(q_{h}\rho_{h}{\mathrm{Cp}}_{h})$—flows steadily and countercurrently within the left- and right-hand subchannels, while the cold feed stream—with time rate of heat capacity $m_{c}\;(q_{c}\rho_{c}{\mathrm{Cp}}_{c})$—flows steadily through the right-hand subchannel.

The principal assumptions are (i) all channel flows are laminar; (ii) fluid physical properties are constant; (iii) the entire MD module is insulated except across the heat-transfer membrane where permeation occurs between the hot-saline and cold feed streams; and (iv) fluid temperatures and velocities are uniform over the cross-sections of the flow channels. Therefore, the total heat-transfer rate may be expressed by

(13) $$\dot{Q}=m_{h}\left(T_{h,i}-T_{h,e}^{0}\right)=m_{c}\left(T_{c,e}-T_{c,i}\right),\;T_{h,i}>T_{c,i}$$

The outlet temperature of the hot-saline feed stream was obtained as

(14) $$T_{h,e}^{0}=T_{h,i}-\frac{m_{c}}{m_{h}}\left(T_{c,e}-T_{c,i}\right)$$

in which $q_{h}$ (or $q_{c}$), $\rho_{h}$ (or $\rho_{c}$) and ${Cp}_{h}$ (or ${Cp}_{c}$) denote the volume flow rates, mass densities and heat capacities of the fluids.

The energy balance over the whole membrane distillation module operated with internal recycle is

(15) $$m_{c}\left(T_{c}{-T}_{c,i}\right)=m_{h}\left(T_{h,i}-T_{h,1}\right)+m_{h}\left(T_{h,2}-T_{h,e}^{0}\right)$$

or the temperature of the cold feed stream is expressed as

(16) $$T_{c}=T_{c,i}+\left(\frac{m_{h}}{m_{c}}\right)\left({T_{h,i}-T}_{h,1}+T_{h,2}-T_{h,e}^{0}\right)$$

**Figure 7:**
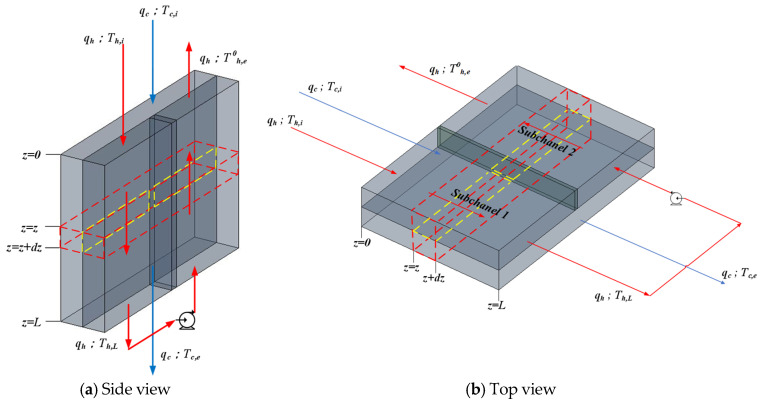
The energy balance made within a finite fluid element (dotted lines) in a flat-plate membrane module.

These temperature polarization coefficients $\gamma_{m,1}$ and $\gamma_{m,2}$ were integrated into one-dimensional governing equations based on macroscopic modeling to solve for temperature variations along the flow length (*dz*), as depicted in Figure 7.

(17) $$-m_{h}\frac{dT_{h,1}}{dz}=\left(\frac{W}{2}\right)H_{m}\gamma_{m,1}\left(T_{h,1}-T_{c}\right)$$

(18) $$m_{h}\frac{dT_{h,2}}{dz}=\left(\frac{W}{2}\right)H_{m}\gamma_{m,2}\left(T_{h,2}-T_{c}\right)$$

where $\gamma_{m,1}$ and $\gamma_{m,2}$ are the temperature polarization coefficients in the left-hand-side and right-hand-side subchannels, respectively, while $H_{m}$ is the overall heat-transfer coefficient of the membrane.

### 3.3. Outlet Temperature

Substituting the value of $T_{c}$ from Equation (16) into Equations (17) and (18), one obtains

(19) $$\frac{dT_{h,1}}{dz}+\alpha T_{h,1}+\alpha^{0}T_{h,2}=\zeta T_{c,e}$$

(20) $$\frac{dT_{h,2}}{dz}+{\beta T}_{h,2}+\beta^{0}T_{h,1}=\xi T_{c,e}$$

where

(21) $$\begin{array}{c} \alpha ={\left(w/2\right)H}_{m}\gamma_{m,1}\left[\frac{\left({m_{c}+m}_{h}\right)}{{m_{h}m}_{c}}\right],\;\alpha^{0}=-\frac{{\left(w/2\right)H}_{m}\gamma_{m,1}}{m_{c}},\;\zeta =\frac{\left(w/2\right)H_{m}\gamma_{m,1}}{m_{h}},\\ \;\;\;\;\;\beta ={\left(w/2\right)H}_{m}\gamma_{m,2}\left[\frac{\left({m_{h}-m}_{c}\right)}{{m_{h}m}_{c}}\right],\;\beta^{0}=-\frac{{\left(w/2\right)H}_{m}\gamma_{m,2}}{m_{c}},\;\xi =-\frac{{\left(w/2\right)H}_{m}\gamma_{m,2}}{m_{h}} \end{array}$$

Equations (19) and (20) can be solved simultaneously for fluid temperatures, $T_{h,1}$ and $T_{h,2}$, in both subchannels in the hot-saline feed side with the following boundary conditions:

(22) $$T_{h,1}=T_{h,2}=T_{h,L}\;\mathrm{at}\;z=L$$

The results are

(23) $$T_{h,1}=c_{1}e^{\lambda_{1}z}{+c}_{2}e^{\lambda_{2}z}+nT_{c,e},\;n=\frac{\beta \zeta -\alpha^{0}\xi }{\alpha \beta -\alpha^{0}\beta^{0}}$$

(24) $$T_{h,2}=c_{3}e^{\lambda_{1}z}{+c}_{4}e^{\lambda_{2}z}+n^{0}T_{c,e},\;n^{0}=\frac{\alpha \xi -\beta^{0}\zeta }{\alpha \beta -\alpha^{0}\beta^{0}}$$

in which two eigenvalues, $\lambda_{1}$ and $\lambda_{2}$, are

(25) $$\lambda_{1}=\frac{-\left(\alpha +\beta \right)+\sqrt{{\left(\alpha -\beta \right)}^{2}+4\alpha^{0}\beta^{0}}}{2},\;\lambda_{2}=\frac{-\left(\alpha +\beta \right)-\sqrt{{\left(\alpha -\beta \right)}^{2}+4\alpha^{0}\beta^{0}}}{2}$$

and where $c_{1}$, $c_{2}$, $c_{3}$ and $c_{4}$ are the integration constants which are determined by Equation (22) as

(26) $$\begin{array}{c} c_{1}=\frac{e^{-\lambda_{1}L}}{\left(\lambda_{2}-\lambda_{1}\right)}\left[-\left({n\lambda }_{2}+\zeta \right)T_{c,e}{+\left(\lambda_{2}+\alpha +\alpha^{0}\right)T}_{h,L}\right],\\ c_{2}=\frac{e^{-\lambda_{2}L}}{\left(\lambda_{1}-\lambda_{2}\right)}\left[-\left({n\lambda }_{1}+\zeta \right)T_{c,e}{+\left(\lambda_{1}+\alpha +\alpha^{0}\right)T}_{h,L}\right],\\ \;\;\;\;\;\;\;\;c_{3}=\frac{-(\lambda_{1}+\alpha )}{\alpha^{0}}c_{1},\;c_{4}=\frac{-(\lambda_{2}+\alpha )}{\alpha^{0}}c_{2} \end{array}$$

Similarly, Equations (23) and (24) can be solved simultaneously for fluid temperatures, $T_{h,i}$ and $T_{h,e}^{0}$, in both holt saline and cold feed streams with the following boundary conditions:

(27) $$T_{h,i}=T_{h,1}\;\mathrm{and}\;T_{h,e}^{0}=T_{h,2}\;\mathrm{at}\;z=0$$

One may obtain $T_{h,i}$ and $T_{h,e}^{0}$ with the introduction of Equations (25) and (26), respectively, and with the substitution of Equation (27), one obtains

(28) $$T_{h,i}=AT_{c,e}+BT_{h,L}$$

(29) $$T_{h,e}^{0}=FT_{c,e}+GT_{h,L}$$

where

(30) $$A=\frac{e^{-\lambda_{1}L}(n\lambda_{2}+\zeta )-e^{-\lambda_{2}L}(n\lambda_{1}+\zeta )}{\left(\lambda_{1}-\lambda_{2}\right)}+n$$

(31) $$B=\frac{{-e}^{-\lambda_{1}L}\left(\lambda_{2}+\alpha +\alpha^{0}\right)+e^{-\lambda_{2}L}(\lambda_{1}+\alpha +\alpha^{0})}{\left(\lambda_{1}-\lambda_{2}\right)}$$

(32) $$F=\frac{1}{\alpha^{0}}\left[\frac{{-e}^{-\lambda_{1}L}\left(\lambda_{1}+\alpha \right)\left(n\lambda_{2}+\zeta \right)+e^{-\lambda_{2}L}\left(\lambda_{2}+\alpha \right)(n\lambda_{1}+\zeta )}{\left(\lambda_{1}-\lambda_{2}\right)}+\left(\zeta -\alpha n\right)\right]$$

(33) $$G=\frac{1}{\alpha^{0}}\left[\frac{e^{-\lambda_{1}L}\left(\lambda_{1}+\alpha \right)\left(\lambda_{2}+\alpha +\alpha^{0}\right)-e^{-\lambda_{2}L}\left(\lambda_{2}+\alpha \right)(\lambda_{1}+\alpha +\alpha^{0})}{\left(\lambda_{1}-\lambda_{2}\right)}\right]$$

### 3.4. Temperature Distributions in Single-Flow Operations

**Figure 8:**
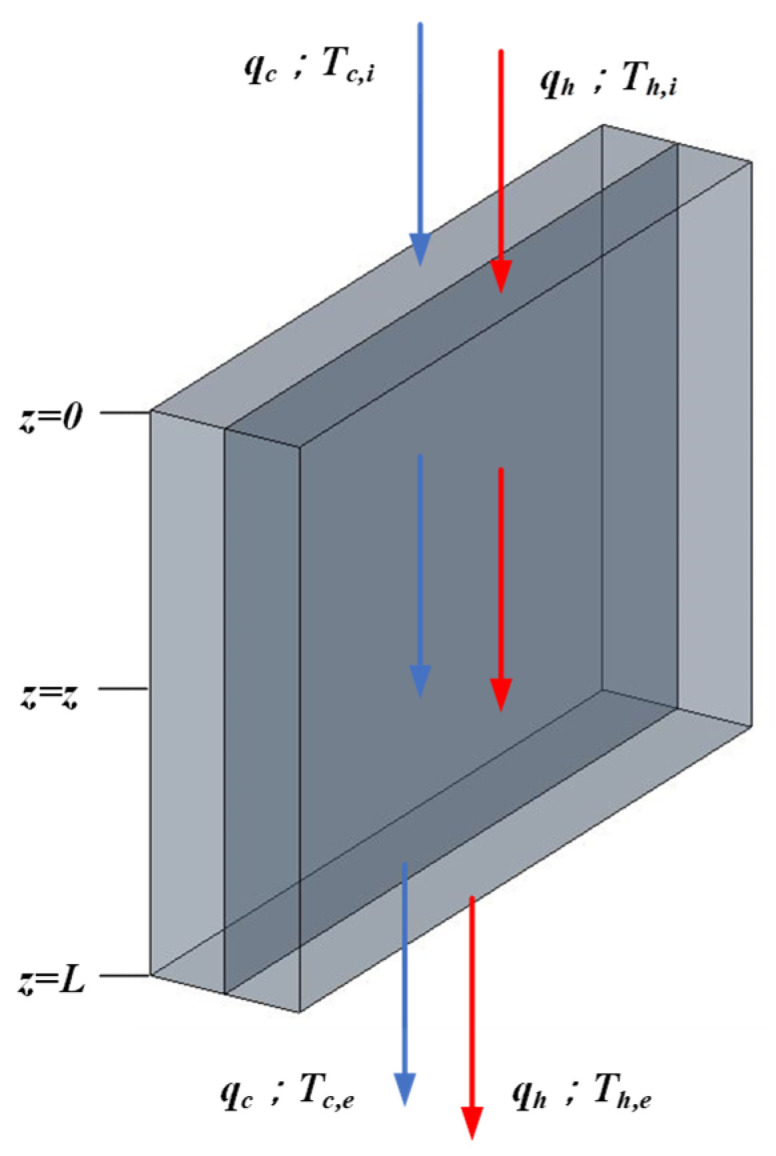
Single-flow device without internal recycle.

**Figure 9:**
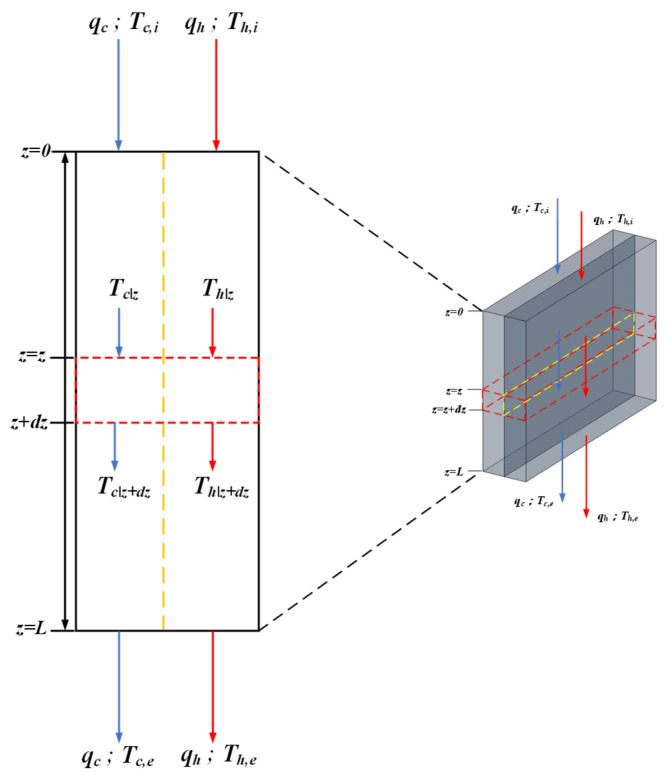
The energy balance made within a finite fluid element in a single-flow module.

The single-flow system whose transfer area and working dimensions are the same as those in the double-flow system and the schematic diagram is shown in Figure 8, while the temperature variations along the flow direction were developed, as illustrated in Figure 9.

The temperature distributions of both streams in terms of the temperature polarization coefficient $\gamma_{m}=\frac{h_{h}h_{c}}{h_{h}h_{c}+h_{h}H_{m}+h_{c}H_{m}}\;$ over the length *dz* are as follows:

(34) $${-m}_{h}\frac{dT_{h}}{dz}={WH}_{m}\gamma_{m}\left(T_{h}-T_{c}\right)$$

or

(35) $$-m_{h}\frac{dT_{h}}{dz}={{WH}_{m}\gamma_{m}\left(T_{h}-T_{c}\right)=WH}_{m}\gamma_{m}\left[T_{h}-T_{c,i}-\left(\frac{m_{h}}{m_{c}}\right)\left({T_{h,i}-T}_{h}\right)\right]$$

The calculation procedure for a single-flow device is much simpler than that for a double-pass device. By following the same mathematical treatment performed in the previous sections of double-flow operatiopns, the results for fluid temperatures are

(36) $$T_{h}\left(z\right)=\left[T_{h,i}-\frac{1}{r}\left(\zeta T_{c,i}+\zeta^{0}T_{h,i}\right)\right]e^{-rz}+\frac{1}{r}\zeta T_{c,i}+\frac{1}{r}\zeta^{0}T_{h,i}$$

and

(37) $$T_{c}\left(z\right)=T_{c,i}+\left(\frac{m_{h}}{m_{c}}\right)\left[{T_{h,i}-T}_{h}\left(z\right)\right]$$

where

(38) $$r=\frac{W\gamma_{m}H_{m}}{m_{h}}+\frac{W\gamma_{m}H_{m}}{m_{c}},\;\zeta =\frac{W\gamma_{m}H_{m}}{m_{h}},\;\zeta^{0}=\frac{W\gamma_{m}H_{m}}{m_{c}}$$

## 4. Results and Discussions

### 4.1. Lessening Temperature Polarization Effect by Operating Double-Flow Channels

**Figure 10:**
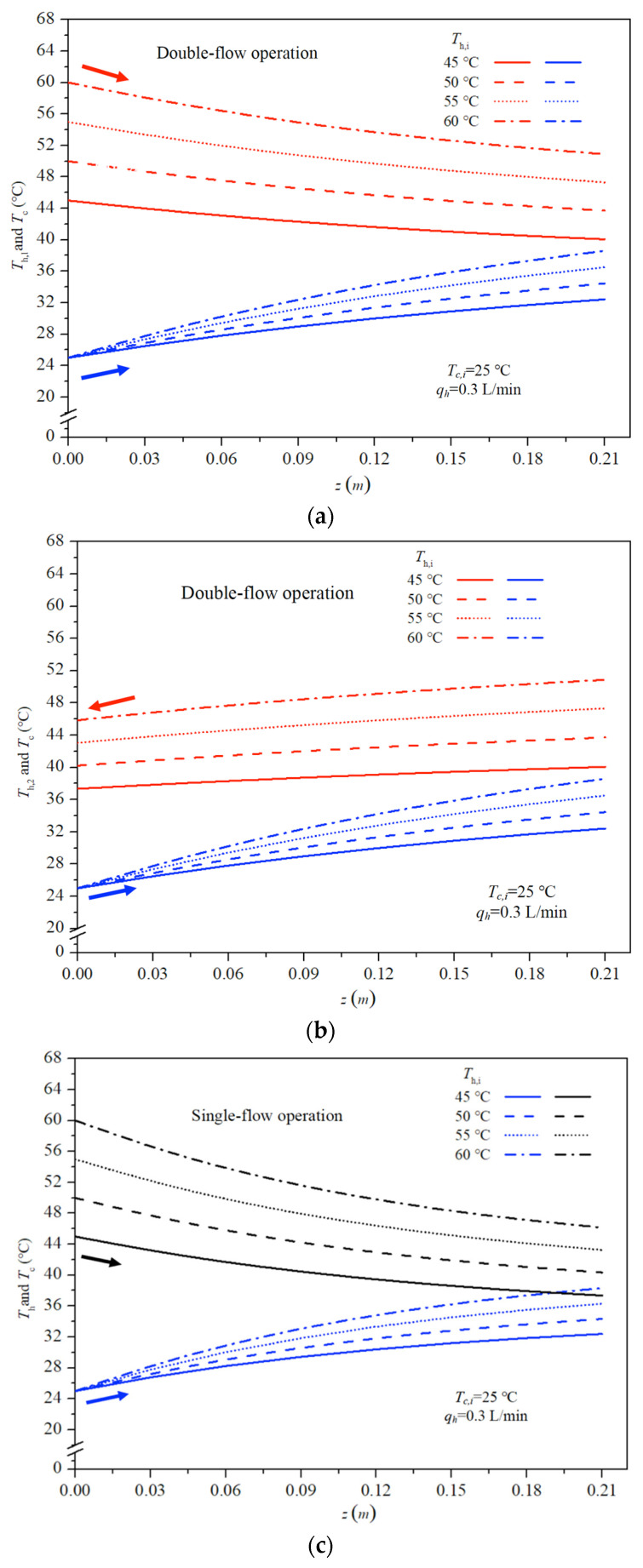
Effects of inlet hot-saline temperatures on temperature distributions along the module. (**a**) $T_{h,1}$ in double-flow operation; (**b**) $T_{h,2}$ in double-flow operation; (**c**) $T_{h}$ in single-flow operation. Red line of the hot saline feed stream for double-flow operation, black line of the hot saline feed stream for single-flow operation and blue line of the cold feed stream.

A one-dimensional theoretical model was used to numerically determine the bulk-temperature distributions of the hot-saline and cold feed streams in the DCMD modules. Figure 10 illustrates these axial profiles for both single- and double-flow operations, parametrized by the hot-saline inlet temperatures of $T_{h,1}$, $T_{h,2}$ and $T_{c}$, respectively. The theoretical predictions indicate that double-flow operation creates tapered bulk temperatures along the flow direction. This results in sharper driving-force temperature gradients across the membrane surfaces compared to single-pass channels, thereby facilitating greater vapor transport and enhancing permeate flux.

### 4.2. The Permeate Flux Improvement

**Figure 11:**
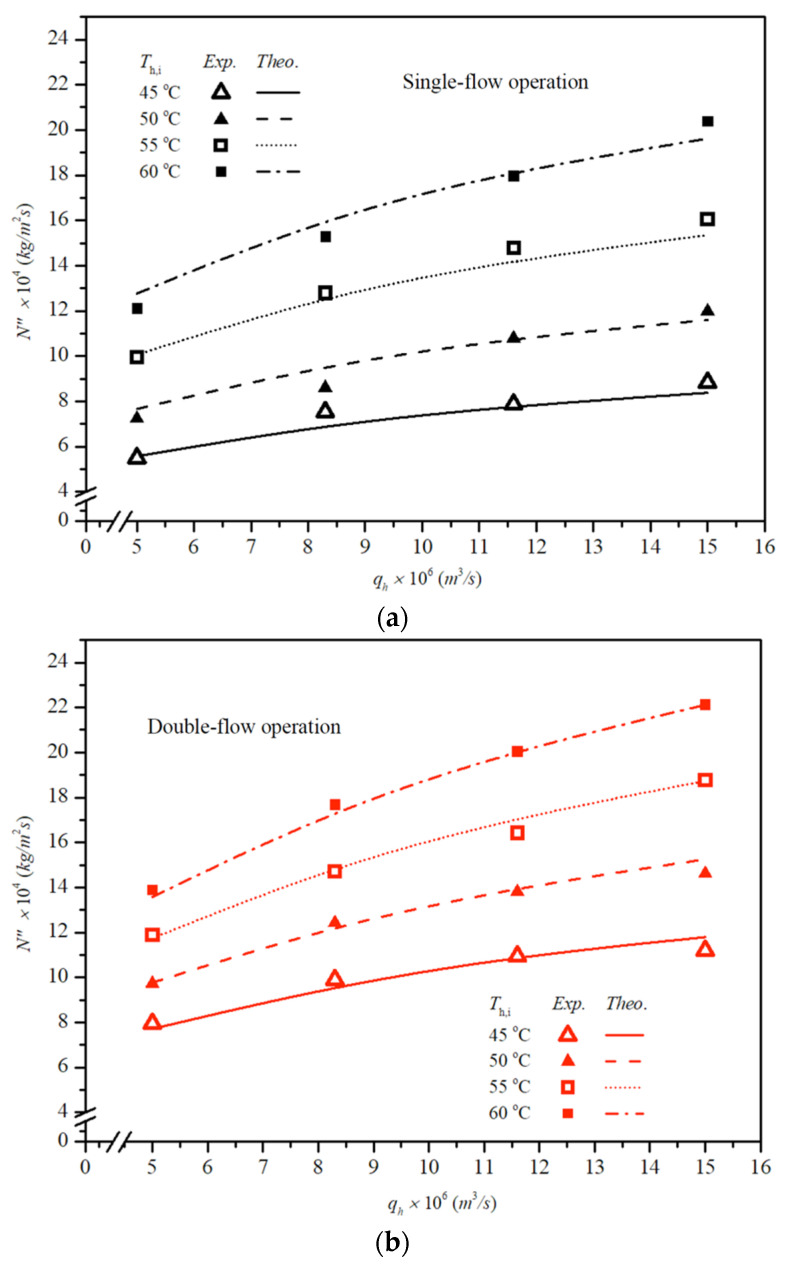
Effect of inlet hot-saline feed temperature and feed flow rate on permeate fluxes. (**a**) Single-flow operation; (**b**) double-flow operation.

The results indicate that higher inlet saline-feed temperatures lead to higher heat-transfer rates. Both experimental findings and theoretical predictions of permeate flux are presented using inlet hot-saline temperatures and feed flow rate as parameters, as shown in Figure 11 for single- and double-flow operations, respectively. The fair consistency and agreement between theory and experiment provide a robust basis for evidence-based validation, as confirmed from Figure 11. Notably, larger permeate fluxes are achieved at higher hot-saline feed temperatures, attributable to a greater saturated vapor-pressure gradient across the membrane.

Both bulk temperature distributions of hot-saline and cold feed streams as well as membrane surface concentrations in membrane absorption modules were solved analytically, as presented in Equations (11) and (12) along the axial coordinate for double-flow operations, and as presented in Equation (24) for single-flow operations. The precision analysis of experimental uncertainty for each individual measurement from the experimental results was determined [35] as follows:

(39) $$S_{N_{exp}^{\prime \prime }}={\left\{\sum_{i=1}^{N_{exp}}\frac{{\left(N_{\exp,i}^{\prime \prime }-\bar{N_{\exp,i}^{\prime \prime }}\right)}^{2}}{N_{exp}-1}\right\}}^{1/2}$$

where the mean value of the resulting uncertainty of the experimental measurements was defined by

(40) $$S_{\bar{N_{exp}^{\prime \prime }}}=\frac{S_{N_{exp}^{\prime \prime }}}{\sqrt{N_{exp}}}$$

The mean experimental uncertainty in Figure 9 ranges between $4.57\times 10^{-3}\;{\leq S}_{\bar{N_{exp}^{\prime \prime }}}\leq 8.37\times 10^{-3}$. Meanwhile, the accuracy deviation between the experimental results and theoretical predictions was calculated as follows:

(41) $$E=\frac{1}{N_{exp}}\sum_{i=1}^{N_{exp}}\frac{\left|N_{theo,i}^{\prime \prime }-N_{\exp,i}^{\prime \prime }\right|}{N_{theo,i}^{\prime \prime }},\;\mathrm{accuracy}\;\mathrm{deviation}$$

where $N_{exp}$*,* $N_{theo,j}^{\prime \prime }$ *and* $N_{exp,j}^{\prime \prime }$ are the number of experimental runs, theoretical predictions and experimental results of absorption fluxes, respectively. The agreement of experimental results deviated from theoretical predictions is well-minimized within $7.0\times 10^{-4}\leq E\leq 1.05\times 10^{-1}$, as shown in Table 1.

The enhanced convective heat-transfer coefficient and enlarged residence time in double-flow operations result in a higher permeate flux. This enhancement, brought about by the implementation of double-flow operations in the hot-saline feed stream, reduces the thermal boundary layer thickness, and consequently, leads to a larger temperature polarization coefficient. Permeate flux was found to increase with higher hot-saline feed flow rates for double-flow configurations; a higher hot-saline feed flow rate induces faster velocities, which effectively reduces heat transfer resistance within the thermal boundary layer. Therefore, the difference in permeate flux performance increases with increasing feed flow rate at constant feed temperature. It is noteworthy that higher permeate flux was achieved at elevated inlet hot-saline temperatures, with the order of flux magnitude as follows: 60 °C > 55 °C > 50 °C > 45 °C. By contrast, the temperature polarization effect is more pronounced in the second channel (the right-hand-side channel) of the module compared to the first channel (the left-hand-side channel). This indicates that permeate flux improvements with operating double-flow modules increase with higher inlet saline temperatures but tend to decrease at higher feed flow rates, at which the difference in permeate flux performance begins to decrease as the inlet hot-saline temperature increases.

It is noteworthy that a higher permeate flux was achieved at a higher inlet saline feed temperature and volumetric flow rate for both single- and double-flow operations. The relative permeate flux improvements $I_{N}$ were illustrated by calculating the percentage increase in comparisons between the permeate flux of the module using single-flow modules and those of double-flow modules, as shown below.

(42) $$I_{N}(\%)=\frac{N_{double}^{\prime \prime }-N_{single}^{\prime \prime }}{N_{single}^{\prime \prime }}\times 100,\;\mathrm{module}\;\mathrm{with}\;\text{double-flow}\;\mathrm{channels}$$

where the subscripts *double* and *single* represent the channels under single-flow and double-flow operations, respectively.

Theoretical predictions of permeate-flux improvements $I_{N}$ for membrane-distillation modules are summarized in Table 1, with inlet hot-saline temperature and feed flow rate as parameters. The analysis reveals that double-flow operation facilitates larger mass diffusion through the membrane, producing higher permeate fluxes than the single-flow operation. The double-flow module exhibits a relative permeate-flux increment of up to 40.77% at an inlet hot-saline temperature of 45 °C and a 0.9 L/min feed flow rate, compared with the single-flow module, as reported in Table 1. 

Furthermore, the double-flow operation causes the permeate flux improvement due to mitigation of the thermal boundary layer with a smaller thermal resistance; therefore, Table 2 represents the thermal resistance reduction under Reynolds numbers within 48.0 $\leq {Re}_{h}\leq 173.8$ and 86.0 $\leq {Re}_{h}\leq 336.5$ for both single- and double-flow operations, respectively. Moreover, the thermal resistance (or convection resistance) was defined as follows:

(43) $$R_{conv}=\frac{1}{h_{h}A}$$

The results show that the thermal resistances decrease with both the inlet hot-saline temperatures and the hot-saline feed flow rates, which is consistent with the decrease of transport resistances by implementing double-flow modules.

### 4.3. Power Consumption Increment

An unavoidable increase in hydraulic consumption—and thus extra energy demand—is expected when operating the double-flow module on the hot-saline feed stream. This increment may be estimated using the Fanning friction factor ($f_{F}$) [32]; for simplicity of making a comparison, the friction losses caused by a joint and a diversion of a bending of a tube are neglected and only wall-friction losses in both the hot and cold feed streams are considered, as follows:

(44) $$H=H_{h}+H_{c}=Q_{h}\rho_{h}lw_{f,h}+Q_{c}\rho_{c}lw_{f,c}$$

(45) $${lw}_{f,h}=\frac{2f_{F,h}{\bar{v}}_{h}^{2}L}{D_{h,h}}$$

The Fanning friction factor can be estimated using a correlation based on the aspect ratio of the channel (α=d/W) [36]:

(46) $$f_{F,h}=\frac{C}{{Re}_{h}},f_{F,c}=\frac{C}{{Re}_{c}}$$

(47) $$C=24\left(1-1.3553\alpha +1.9467\alpha^{2}-1.7012\alpha^{3}+0.9564\alpha^{4}-0.2537\alpha^{5}\right)$$

The percentage of the relative extents $I_{P}$ of the energy consumption increment for operating double-flow modules is illustrated in comparison to the single-flow module as

(48) $$I_{P}=\frac{H_{double}-H_{single}}{H_{single}}\times 100\%$$

**Figure 12:**
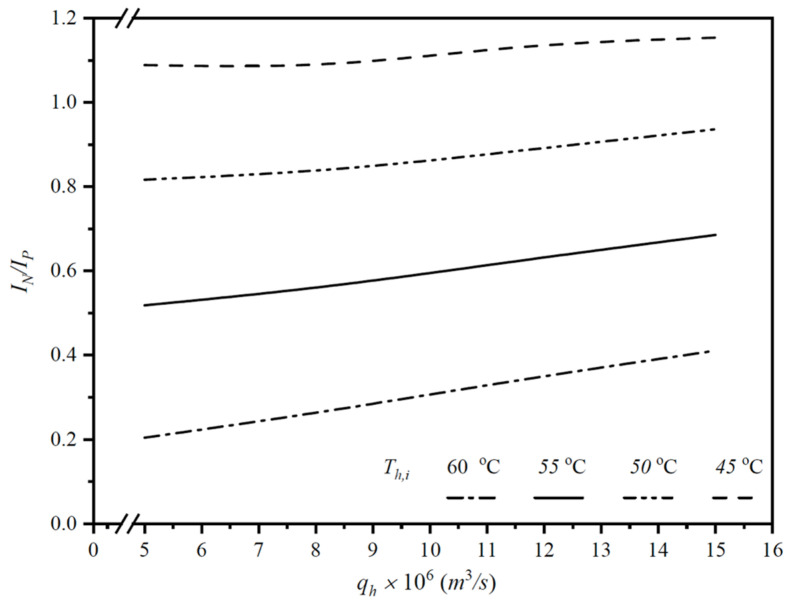
Comparisons of $I_{N}/I_{P}$ with employing double-flow operations under inlet hot-saline temperatures.

The appropriate selection of operating conditions that balance permeate-flux enhancement against the increase in energy consumption was assessed from an economic viewpoint for the double-flow modules, represented by the ratio $I_{N}/I_{P}$. The technical feasibility, accounting for the trade-offs of additional friction losses, was examined accordingly. A higher $I_{N}/I_{P}$ ratio can be achieved by suitably employing the double-flow configuration, enabling more effective permeate-flux gains at the expense of energy consumption, as shown in Figure 12, with inlet hot-saline temperatures and flow rates as parameters.

Notably, the double-flow channels induce higher turbulence intensity, resulting in enhanced mass-transfer coefficients. The comparison reveals that a higher permeate flux $I_{N}$ is achieved when operating at higher inlet hot-saline temperatures and volumetric flow rates, as shown in Table 1. By contrast, the ratio $I_{N}/I_{P}$ favors operation at lower inlet hot-saline temperatures, as shown in Figure 12. In other words, when evaluated economically, double-flow channels can increase permeate flux more effectively at lower inlet hot-saline temperatures, given the associated increase in energy consumption. In summary, the percentage increase in permeate-flux improvement exceeded the percentage increase in energy consumption. Essentially, operating double-flow channels in the hot-saline feed achieved desirable permeate-flux enhancement while counterbalancing the undesirable increase in friction losses. 

## 5. Conclusions

The recent DCMD literature confirms the technology’s potential for sustainable desalination, with research moving beyond simple feasibility studies towards targeted performance optimization. The shift toward recycle-enhanced and hybrid system configurations highlights a clear focus on improving overall energy efficiency and achieving high water recovery from challenging feed waters. It is concluded that considerable improvement in permeate flux is obtainable when membrane distillation is operated with internal recycle arranged in the hot-saline feed stream. The temperature distributions and permeate flux derived from the theoretical model provide valuable guidance for designing more efficient membrane-distillation modules. Moreover, the double-flow device (with internal recycle) provides the desirable effects of increased fluid velocity and an enhanced heat-transfer coefficient, resulting in higher permeate flux—especially at larger inlet hot-saline feed temperatures. Thorough comparisons of permeate-flux improvement by operating double-flow channels lead to the following conclusions:

1. Operating double-flow channels in the hot-saline feed stream yields relative increases in permeate flux, with a maximum improvement of 40.77% at an inlet hot-saline temperature of 45 °C and a 0.9 L/min hot-saline feed flow rate, compared with a single-flow channel module.
2. The results demonstrate that double-flow channels in membrane-distillation modules achieve a more pronounced permeate-flux improvement $I_{N}$ due to a larger convective heat-transfer coefficient.
3. The study shows higher permeate-flux improvement $I_{N}$ in modules with larger inlet hot-saline temperatures; however, the ratio $I_{N}/I_{P}$ for double-flow channels follows a reverse order.

The present paper is actually the extension of another recycle problem in previous works [27,28], except that the membrane distillation modules are introduced to improve the permeate flux. Figure 11 illustrates some results obtained from the single-pass operations for comparisons. The advantage of the present double-flow-channel module is evident. While this study focuses on evaluating permeate-flux improvement and energy-consumption increment by operating a double-flow module on the hot-saline feed channel, from an economic viewpoint, further investigation is warranted to explore recycling double-pass channels with recycle ratio optimization.

## Figures and Tables

**Table 1:** The accuracy deviation between theoretical predictions $N_{theo}^{\prime \prime }\;$ and experimental results $N_{exp}^{\prime \prime }\;$ of permeate fluxes.

$T_{h,in}$ (°C)	$q_{h}$ L/min	Single-Flow Operations			Double-Flow Operations			
		$N_{exp}^{\prime \prime }\times 10^{4}$ (kg m^−2^ s^−1^)	$N_{theo}^{\prime \prime }\times 10^{4}$ (kg m^−2^ s^−1^)	E (%)	$N_{exp}^{\prime \prime }\times 10^{4}$ (kg m^−2^ s^−1^)	$N_{theo}^{\prime \prime }\times 10^{4}$ (kg m^−2^ s^−1^)	E (%)	$I_{N}$ (%)
45	0.3	5.50	5.57	1.32	7.95	7.71	3.13	38.42
	0.5	7.53	6.97	8.12	9.90	9.63	2.83	38.21
	0.7	7.87	7.80	0.92	10.94	10.94	0.07	40.24
	0.9	8.83	8.37	5.48	11.20	11.79	4.95	40.77
50	0.3	7.24	7.66	5.57	9.73	9.77	0.40	27.47
	0.5	8.60	9.62	10.54	12.43	12.32	0.88	28.15
	0.7	10.80	10.79	0.08	13.81	14.00	1.41	29.81
	0.9	11.98	11.60	3.31	14.62	15.25	4.15	31.50
55	0.3	9.96	10.06	1.06	11.89	11.74	1.25	16.69
	0.5	12.79	12.68	0.89	14.71	14.97	1.78	18.10
	0.7	14.79	14.25	3.76	16.42	17.12	4.10	20.14
	0.9	16.07	15.35	4.68	18.78	18.74	0.26	22.07
60	0.3	12.12	12.77	5.06	13.89	13.58	2.28	6.32
	0.5	15.29	16.15	5.32	17.68	17.49	1.10	8.29
	0.7	17.98	18.20	1.20	20.03	20.13	0.46	10.62
	0.9	20.39	19.62	3.94	22.13	22.12	0.07	12.72

**Table 2:** Thermal resistances of modules for both single- and double-flow operations.

$T_{h,i}$ (°C)	$q_{h}$ (L/min)	$\mathrm{Thermal}\;\mathrm{Resistances}\times 10^{2}$ (K/W)	
		Single Flow	Double Flow
45	0.3	2.28	2.20
	0.5	2.24	2.11
	0.7	2.19	2.03
	0.9	2.15	1.96
50	0.3	2.27	2.18
	0.5	2.22	2.10
	0.7	2.17	2.01
	0.9	2.13	1.94
55	0.3	2.25	2.16
	0.5	2.20	2.08
	0.7	2.16	2.00
	0.9	2.11	1.93
60	0.3	2.23	2.15
	0.5	2.19	2.06
	0.7	2.14	1.99
	0.9	2.10	1.92

## Data Availability

The original contributions presented in this study are included in the article. Further inquiries can be directed to the corresponding author.

## Nomenclature

A	Cross-sectional area of flow channel (m^2^)
$a_{w}$	Water activity in NaCl solution
C	Friction losses coefficient
$C_{P,j}$	Heat capacity ($J\;kg^{-1}\;K^{-1}$), j=1,2
$c_{k}$	Membrane coefficient based on the Knudsen diffusion model ($\mathrm{mol}\;m^{-2}Pa^{-1}s^{-1}$)
$c_{M}$	Membrane coefficient based on the molecular diffusion model ($\mathrm{mol}\;m^{-2}Pa^{-1}s^{-1}$)
$c_{m}$	Membrane permeation coefficient ($\mathrm{mol}\;m^{-2}Pa^{-1}s^{-1}$)
d	Channel height (m)
$D_{m}$	Diffusion coefficient of air and vapor in the membrane ($m^{2}s^{-1}$)
${De}_{h,j}$	Hydraulic equivalent diameter of channel (m), j=h, c
E	Accuracy deviation of experimental results from the theoretical predictions
$f_{F,c}$	Fanning friction factor for cold feed stream
$f_{F,h}$	Fanning friction factor for hot-saline feed stream
H	Hydraulic dissipate energy (W)
$I_{N}$	Permeate flux relative factor
$I_{p}$	Power consumption relative index
L	Channel length (m)
$lw_{f,j}$	Friction loss (J kg^−1^), j=h,c
$M_{W}$	Molecular weight of water (kg mol^−1^)
$m_{c}$	Cold feed stream with time rate of heat capacity (J K^−1^ s^−1^)
$m_{h}$	Hot-saline feed stream with time rate of heat capacity (J K^−1^ s^−1^)
$N^{\prime \prime }$	Distillate flux ($\mathrm{kg}\;m^{-2}s$)
$\bar{N^{\prime \prime }}$	Average value of $N^{\prime \prime }$ for all number of experimental measurements ($\mathrm{kg}\;m^{-2}s$)
$N_{exp}$	Number of experimental measurements
$P_{m}$	Mean saturated pressure in membrane (Pa)
$P^{sat}$	Saturation vapor pressure (Pa)
$q_{j}$	Volumetric flow rate (m^3^ s^−1^), j=h,c
$q^{\prime \prime }$	Heat flux ($J\;m^{-2}s^{-1}$)
r	Membrane pore radius (m)
R	Gas constant (8.314 J mol^−1^ K^−1^)
Re	Reynolds number
$S_{N^{\prime \prime }}$	Uncertainty of the experimental measurements
$S_{\bar{N^{\prime \prime }}}$	Mean value of $S_{N^{\prime \prime }}$
$T_{cm}$	Membrane surface temperature in the cold feed region (K)
$T_{hm}$	Membrane surface temperature in the hot-saline feed region (K)
$T_{m}$	Mean temperature in membrane (K)
$\bar{v}$	Average velocity ($m\;s^{-1}$)
W	Width of channel (m)
$x_{w}$	Liquid mole fraction of water
$x_{\mathrm{NaCl}}$	Mole fraction of NaCl in saline solution
$Y_{w}$	Vapor mole fraction of water
${\left|Y_{m}\right|}_{ln}$	Natural log mean vapor mole fraction of water in the membrane
z	Axial coordinate along the flow direction (m)
*Greek letters*	
$\delta_{m}$	Thickness of membrane (µm)
ε	Membrane porosity
$\eta_{v}$	Gas viscosity ($\mathrm{Ns}\;m^{-2}$)
λ	Latent heat of water ($J\;kg^{-1}$)
$\lambda_{1},\lambda_{2}$	Eigenvalues
μ	Viscosity ($\mathrm{Ns}\;m^{-2}$)
ρ	Density ($\mathrm{kg}\;m^{-3}$)
$\gamma_{m}$	Temperature polarization coefficients
τ	Membrane tortuosity
*Subscripts*	
1	Channel 1 of the hot-saline feed side
2	Channel 2 of the cold feed side
*c*	Cold feed stream
*double*	Double-flow operation
*h*	Hot feed stream
*single*	Single-flow operation
*exp*	Experimental results
*i*	At the inlet
*e*	At the outlet
*theo*	Theoretical predictions
